# Symptomatic cardiac metastases of breast cancer 27 years after mastectomy: a case report with literature review - pathophysiology of molecular mechanisms and metastatic pathways, clinical aspects, diagnostic procedures and treatment modalities

**DOI:** 10.1186/1477-7819-11-14

**Published:** 2013-01-23

**Authors:** Darko Katalinic, Ranka Stern-Padovan, Irena Ivanac, Ivan Aleric, Damir Tentor, Nora Nikolac, Fedor Santek, Antonio Juretic, Stjepko Plestina

**Affiliations:** 1 Department of Oncology, University Hospital Centre (KBC Zagreb), University of Zagreb School of Medicine, Kispaticeva 12, HR-10000, Zagreb, Croatia; 2 Department of Diagnostic and Interventional Radiology, University Hospital Centre (KBC Zagreb), University of Zagreb School of Medicine, Zagreb, Croatia; 3 Department of Cardiovascular Diseases, University Hospital Centre (KBC Zagreb), University of Zagreb School of Medicine, Zagreb, Croatia; 4 Department of Pulmonary Medicine, University Hospital Centre (KBC Zagreb), University of Zagreb School of Medicine, Zagreb, Croatia; 5 Department of Pathology and Cytology, University Hospital Centre (KBC Zagreb), University of Zagreb School of Medicine, Zagreb, Croatia; 6 Department of Clinical Chemistry, University Hospital Centre “Sisters of Charity”, University of Zagreb School of Medicine, Zagreb, Croatia

**Keywords:** Heart metastases, Breast cancer, Pathophysiology, Symptoms, Treatment

## Abstract

Metastases to the heart and pericardium are rare but more common than primary cardiac tumours and are generally associated with a rather poor prognosis. Most cases are clinically silent and are undiagnosed *in vivo* until the autopsy. We present a female patient with a 27-year-old history of an operated primary breast cancer who was presented with dyspnoea, paroxysmal nocturnal dyspnoea and orthopnoea. The clinical signs and symptoms aroused suspicion of congestive heart failure. However, the cardiac metastases were detected during a routine cardiologic evaluation and confirmed with computed tomography imaging. Additionally, this paper outlines the pathophysiology of molecular and clinical mechanisms involved in the metastatic spreading, clinical presentation, diagnostic procedures and treatment of heart metastases. The present case demonstrates that a complete surgical resection and systemic chemotherapy may result in a favourable outcome for many years. However, a lifelong medical follow-up, with the purpose of a detection of metastases, is highly recommended. We strongly call the attention of clinicians to the fact that during the follow-up of all cancer patients, such heart failure may be a harbinger of the secondary heart involvement.

## Background

Metastases to the heart assume greater diagnostic and therapeutic importance as the incidence of different cancer types rises. The condition was first described by Theophy Boneti in 1700 [1]. Primary tumours of the heart are rare, occurring at a frequency of <0.02% in different autopsy series [2]. However, secondary or metastatic tumours of the heart are more common than primary tumours [3] and patients may present with various cardiac symptoms. Common primary tumours to metastasise to the heart are mediastinal tumours, lung cancer, melanoma, breast cancer and esophageal cancer [4]. Intracavitary growth of secondary heart tumours, however, is quite unusual. Therefore, despite their frequency, metastases to the heart rarely gain clinical attention. The signs of cardiac involvement are often overlooked, since the symptoms of a metastatic disease prevail. We present a case of breast cancer with symptomatic heart metastases and heart chambers involvement 27 years after mastectomy and discuss the pathophysiology of molecular mechanisms of cardiac metastases, metastatic pathways, clinical manifestations, diagnostic procedures and treatment.

## Case presentation

**Figure 1:**
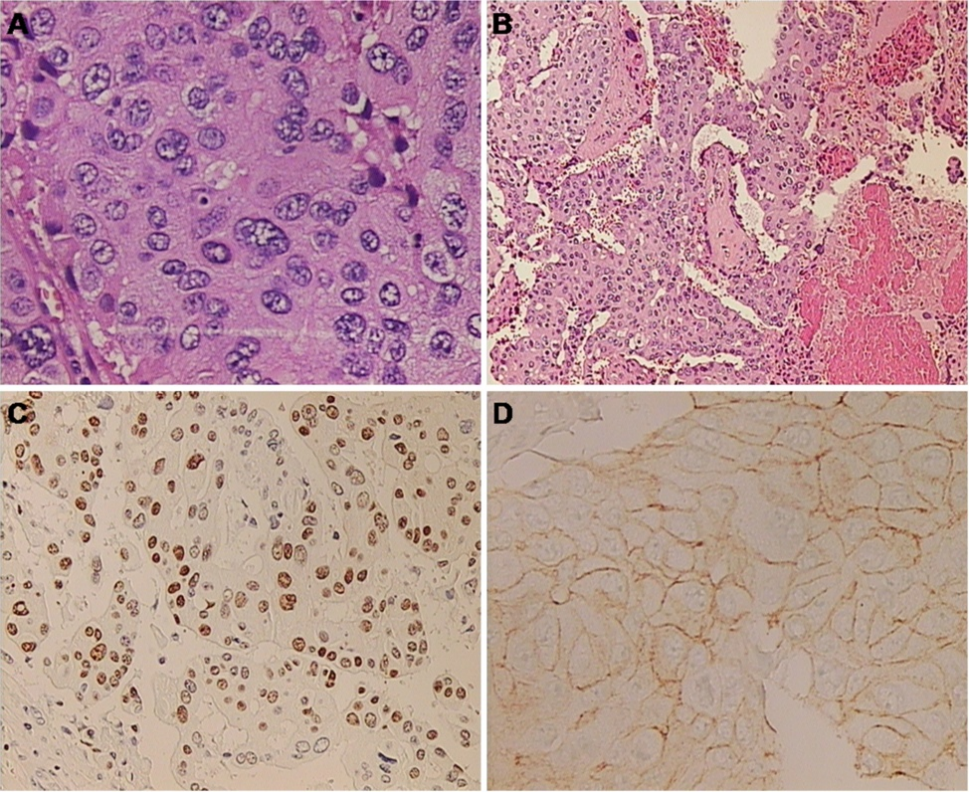
**Histopathological evaluation.** Hematoxylin and eosin (HE) histologic analysis revealed a highly cellular malignant tumour with solid clusters of atypical, polymorphic epithelial cells (image **A**, high-power photomicrograph, original magnification, ×400; HE stain). The tumour was partially necrotic (image **B**, low-power photomicrograph, original magnification, ×100; HE stain). Immunohistochemical study shows that the tumour cells stained positive for estrogene receptors (ER) (image **C**, low-power photomicrograph, original magnification, ×200) and HER-2/neu receptors (image** D**, low-power photomicrograph, original magnification, ×200) consistent with diagnosis of breast adenocarcinoma.

The patient’s past medical history was significant for a right-sided total mastectomy for carcinoma in 1984 (PHD: Adenocarcinoma of the breast) (Figure 1A and 1B). The patient was staged as T2N0M0. At that time, she had no radiation or chemotherapy. All lymph nodes were negative. In 1992, the patient was diagnosed with lung metastases and treated with CMF regimen (cyclophosphamide, methotrexate and fluorouracil), with a partial tumour regression. In 1995, she underwent the ablation of solitary metastasis in the left lung lobe (PHD: Metastatic adenocarcinoma of the breast). In 1999, a complete clinical re-evaluation was made since the hymoptysis and a solitary metastatic tumour in the distal left lung were diagnosed. She underwent a left-sided pulmectomy and mediastinal lymphadenectomy (PHD: Metastatic adenocarcinoma of the breast) with good clinical outcome and the disease was stabile for the following 6 years. In 2005, at regular oncologic follow-up, the patient was diagnosed with a tumour formation under the left scapula which was surgically removed (PHD: Metastatic adenocarcinoma of the breast, ER+, HER-2/neu +++) (Figure 1C and 1D). After that, she was treated with anastrosol and trastuzumab with regular medical follow-up and she felt quite well.

**Figure 2:**
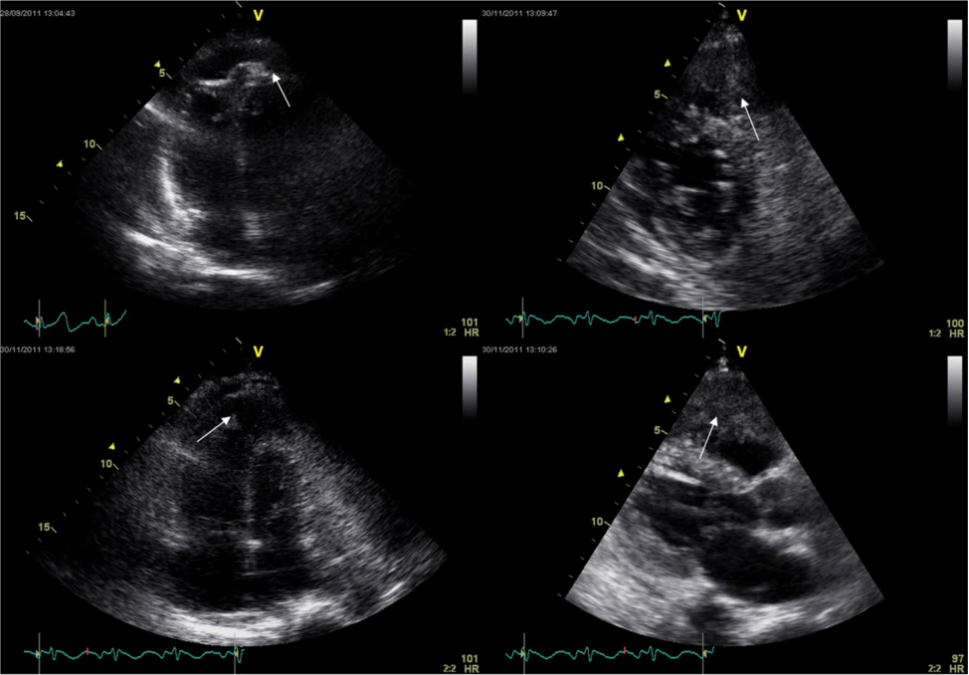
**Echocardiographic evaluation.** Four-chamber 2-dimensional transthoracic echocardiogram shows a large, irregular metastatic mass (2.3×1.1 cm), which infiltrated pericardium and myocardium (predominantly anteroapical and lateral walls of the left ventricle) with intracavitary propagation (arrows).

**Figure 3:**
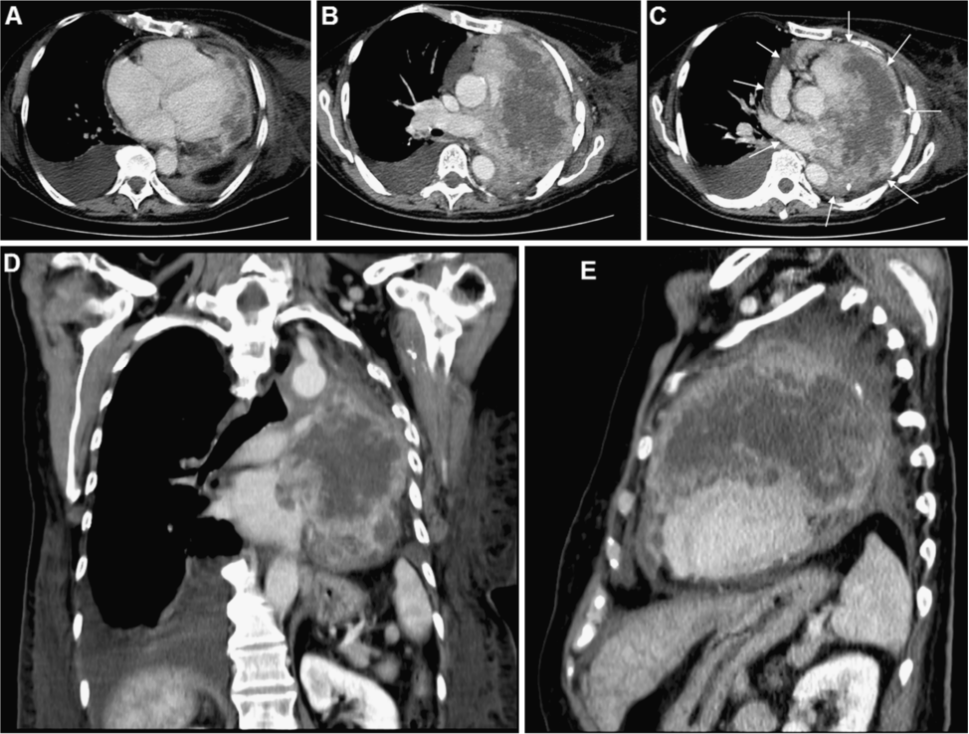
**Radiological evaluation.** Contrast-enhanced axial (Figures 3**A**-3**C**), coronal (Figure 3**D**) and sagittal (Figure 3**E**) multislice computed tomography scan of the chest revealed massive, predominantly necrotic metastatic tumour mass (13×10 cm in size on axial view) with pericardial and heart involvement (arrows). The tumour was partially necrotic and infiltrated the thoracic aorta and the pulmonary trunk with extension into the left atrium.

In 2011, 27 years after mastectomy, a 64-year-old white woman was admitted to our hospital with a 2-week history of shortness of breath, dyspnoea, paroxysmal nocturnal dyspnoea and orthopnoea without chest pain. The clinical presentation aroused suspicion of a congestive heart failure. The Karnofsky’s index of performance status (KPS) was 20% and the Eastern Cooperative Oncology Group Performance Status Scale (ECOG PS) was grade 4. On admission, the patient was afebrile and in a moderate degree of respiratory distress. There was no clinical or laboratory evidence for any kind of inflammation or pulmonary embolism. Her pulse was 140 beats per minute and regular, and her blood pressure was 130/90 mmHg. The heart sounds were distant and faint, there were no heart murmurs. Chest radiography revealed an increase in cardiac silhouette and the lung congestion. The electrocardiogram showed sinus tachycardia and low voltage without other abnormalities. All cardiac enzymes were negative. However, the high values of carbohydrate antigen 15–3 (Ca 15–3) (335.2 kIU/L, normal rate <25) and carcinoembryonic antigen (CEA) (380.8 μg/L, normal rate <3.4) suggested clinical relapse. Two-dimensional transthoracic echocardiography (2D-TTE) revealed cardiac involvement with metastatic tumour mass (Figure 2). A contrast-enhanced multislice computed tomography (MSCT) of the chest confirmed and demonstrated a large metastatic mass that involves predominantly the pericardium and the left ventricle with the extension into the left atrium (Figure 3A-3E). The patient was treated with corticosteroids, amynophilline, digitalis, beta-adrenergic antagonists, analgetics, low-molecular-weight heparin and oxygenation. The palliative radiation therapy was initiated, but because of patient’s severe general condition, a total dose of only 6 Gy/day was delivered as a palliative thoracic radiotherapy. Unfortunately, soon after hospital admission, the patient died. The immediate cause of death was cardiorespiratory arrest due to massive involvement of the heart by metastatic malignancy.

## Discussion

Malignant tumours metastasizing to the heart are one of the least investigated topics in clinical and experimental oncology. In the past, the low incidence of cancer to the heart has been explained on the theory that the heart was not receptive to tumour cells. Prichard mentioned the kneading action and metabolic peculiarity of the heart muscle, and the rapid blood flow as factors accounting for the low incidence [5]. Other authors considered that the low incidence was more likely the result of inadequate observation. Until the 1960s, primary and especially secondary cardiac tumours were merely a curiosity and the diagnosis was academic. Generally, they have devastating consequences given that they involve such an important organ and present in very subtle ways causing a subsequent delay in clinical diagnosis. In the past two decades, imaging has improved detection, with 2D-TTE, 2-dimensional transesophageal echocardiography (2D-TEE), computed tomography (CT) and magnetic resonance imaging (MRI) being the mainstay of clinical evaluation [6]. Because of its easy availability, 2D-TTE, 2D-TEE and radiology procedures such as CT or MRI are complementary techniques, and are all used in the evaluation of cardiac deposits. With the gradual improvement in surgery, chemotherapy, radiotherapy, biotherapy and molecular targeted therapy, life-threatening sites of metastases are more commonly receiving intensive local therapy for palliation. Such tumours are frequently highly responsive to radiotherapy or chemotherapy, and premortem diagnosis may be critical in the clinical management. Although no malignant tumours are known which diffuse preferentially to the heart, some do involve the heart more often than others, for instance lung cancer, breast and esophageal cancer, lymphoproliferative malignancies, as well as melanoma [5,7-12]. Metastasis is cancer’s most deadly aspect and it is affected by several interconnected processes. Therefore, tumour’s ability to spread to the heart tissue depends on many different factors, not only the molecular, biological and pathophysiological characteristics of the primary tumour, but also the specific structural and functional characteristics of the heart [5].

### Pathophysiology of cardiac metastases, metastatic pathways and cascades: from molecular to clinical aspects

Although primary cardiac tumours are extremely rare (different postmortem studies reported rates between 0.001% and 0.28%), secondary tumours are not, and the heart can be metastasised by any sort of malignancy [13,14]. Only tumours of the central nervous system have not been proven to develop heart metastases. The exact incidence of cardiac metastases is unknown. Indeed, secondary deposits are considered rare, but when sought for, the incidence seems to be not as low as expected [15] and they exist usually in the setting of a widely disseminated disease [12]. Reflecting the age distribution, heart metastases predominantly occur in patients between the sixth and eighth decades of life and there is no gender preference. They were found in 1.23% of 12,485 consecutive autopsies, compared with a 0.056% prevalence of primary cardiac tumours [12]. In autopsies at which a malignant neoplasm was diagnosed, cardiac metastases were found in 9.7% to 10.7% of cases [4,16,17]. According to Bussani *et al*., the highest rates of heart metastasis were found in patients diagnosed with pleural mesothelioma (48.4%), melanoma (27.8%), lung cancer (21%) and breast cancer (15.5%). High rates have also been found in patients with ovarian cancer (10.3%) and lymphoproliferative malignancies (9.4%). About two-thirds of all heart metastases involved the pericardium and only one-third the epicardium or the myocardium [18]. Some tumours colonize a wide variety of organs and the first site encountered will be the most common site of metastatic formation. Others are more selective, they bypass some organs and selectively colonize specific distal tissues. Interestingly, for reasons which are not clear, melanoma has a particular predilection for metastasising to the heart and brain and a half of all cases of disseminated melanoma patients will have cardiac deposits at necropsy [19]. However, breast cancer primarily metastasizes to the bone, lungs, regional lymph nodes, liver and brain, with the most common site being the bone.

In 1889, Stephen Paget noticed that metastases in breast cancer were not random. He postulated ‘seed and soil’ hypothesis claiming that a metastatic cells arose from the proliferation of a few tumour cells (the seeds) in the favourable tissue environment provided by certain organs (the soil) [20]. According to the ‘seed and soil’ theory, it is difficult for cancer cells to survive outside their region of origin, so in order to metastasize they must find a location with similar characteristics [20]. For instance, melanoma spreads to the brain, presumably because neural tissue and melanocytes arise from the same cell line in the embryo. A few decades later, in 1928, opposite to Pageth’s opinion, James Ewing challenged the ‘seed and soil’ theory and proposed that metastasis occurs purely by anatomic and mechanical routes [21]. Today, it is widely known that both Paget and Ewing were right and that the two theories are not mutually exclusive. Additionally, organ-specific anatomic considerations also influence the metastatic process. These include blood-flow patterns from the primary tumour and the homing ability of cancer cells to certain tissues and organs. Typical environmental barriers in a metastatic event include physical (a basement membrane - BM), chemical (low pH, reactive oxygen species, hypoxia, and so on) and biological (immune surveillance, inhibitory cytokines and regulatory extra-cellular matrix peptides) components [22]. During tumour invasion, malignant cells penetrate a variety of extracellular matrices (ECM), including BMs and interstitial stroma through multiple steps in the metastatic cascade. To be completely successful, a tumour cell must invade and degrade connective host tissue (invasion of the primary tumour border - BM and the tissue surrounding the tumour by the cell) and subsequently intravasate (the bloodstream or lymph channels), survive the transit into the new environment, arrest in distant vascular bed, extravasate to a distant site (the tumour cell than invades into the BM of the targeted tissue interstitium), initiate a micrometastasis and proliferate to metastatic colony [21,22]. The initial part of this cascade also known as ‘epithelial to mesenchymal transition’ (EMT) was first conceived by Krug *et al*. in 1987 and described by Hay in 1995 [23,24]. It is defined as the switch from non-motile, polarized epithelial cells to motile, non-polarized mesenchymal cells, with the potential to migrate from primary tumour sites to distant tissues and organs, where they can proliferate [23-25]. There are several EMT components, and the most important are E-cadherin, N-cadherin, and transforming growth factor-β (TGF-β) [26]. A ringleader of EMT is a process named the ‘cadherin switch’, defined by a decrease in E-cadherin, the component of adherent junctions, and a simultaneous increase of mesenchymal N-cadherin. This switch allows cells to lose adhesive affinity for other surrounding cells and become more migratory and invasive [26-29]. Another most potent inducer of EMT is the TGF-β, a known pluripotent growth factor, able to induce EMT in mammary and other cell types [26,30,31]. As we know, the process of metastatic spreading is a complex series of different steps. Malignant cells break away from the primary tumour and degrade proteins that make up the surrounding ECM, which separates the tumour from adjoining tissue. Cell-ECM adhesion, motility, and localised proteolysis are mediated mainly by matrix metalloproteases (MMPs). The tumour cell develops structures called invadopodia, which are highly concentrated in several MMPs [32,33]. Therefore, the degradation of ECM is facilitated by MMPs, and tumour cells can move across tissues into nearby stroma. ECM-tumour cell interactions play a critical role in each of the events of the metastatic cascade. Interactions of the breast cancer cells with integrins, fibronectin, tenascin C, laminins, collagens, endoglin, hyaluronan, heparanase and proteoglycans can contribute to the metastatic process. The ECM protein Tenascin C (TNC) is an adhesion-modulating extracellular matrix glycoprotein and it is upregulated in metastatic breast cancer. TNC is highly expressed in tumour stroma and stimulates tumour-cell proliferation. Endoglin is a cell-surface disulfide-linked homodimeric glycoprotein which binds to integrins and is a co-receptor for TGF-β. For instance, brain and heart-metastatic breast-tumour cells also express endoglin in large amounts [34,35]. Heparanase expressed by breast cancer cells participates in angiogenesis and neovascularisation by degrading the polysaccharide scaffold of the endothelial BM, thereby releasing angiogenic growth factors from the ECM [36]. There are also several different cell types critical for tumour growth. Gao *et al*. and Nolan *et al*. demonstrated that endothelial progenitor cells are critical for metastasis and angiogenesis [37,38]. Furthermore, the ablation of endothelial progenitor cells in the bone marrow leads to a significant decrease in tumour growth and vasculature development [39]. Metastatic spreading is also mediated by numerous growth factors and cytokines, operating through different cell signalling pathways. To initiate transcriptional responses, these signalling molecules must enter the nucleus through the nuclear pore complex (NPC). The NPCs are made of proteins named nucleoporins (Nups) and there are several Nups that are often abnormal in cancer patients. Some of them are Ribonucleic acid exports 1 (Rae1) linked to breast cancer pathophysiology [40] and Nup88 which is overexpressed in ovarian cancer [41] as well as in a broad spectrum of sarcomas, lymphomas and mesotheliomas [42,43]. The transport of different cytokines, transcription factors (HER2, TGF-β, galectin-3, NF-κB and other), or metastasis activators across the nuclear envelope commonly requires transporters (karyopherins) that bring them to the nuclear pore. Finally, these molecules are translocated into the nucleus, where they can bind to target genes to initiate transcription and consequently cell migration and proliferation. For the metastasis to occur, all steps in the metastatic cascade must take place. Therefore, the blockade of any single step in the metastatic pathway should slow down metastasis progression [44].

Heart metastases are usually small and multiple; infrequently, single large tumour lesions are also observed. Clinically, they occur by four main routes: transcoelomic, lymphatic, haematogenous and thru implantation. Metastases may reach the heart via the hematogenous or lymphatic route, or by direct or transvenous extension. Lymphatic spread is most important and tends to give rise to pericardial metastases, while hematogenous spread preferentially gives rise to myocardial metastases. Tumours such as the breast cancer, which develop near the heart, may expand by direct extension into the heart tissue, but these tumours predominantly attain to the heart by the lymphatics. Via mediastinal lymph nodes, tumour cells intially invade the epicardial, and then the myocardial lymphatic system [45]. As in our case, secondary heart tumours that have partial or total intracavitary growth are rare; when they do occur, they are often accompanied by thrombosis [46-51].

The structure of the lymphatic system in the heart might explain the low incidence of secondary deposits in the heart compared with other tissues and organs [18]. As a particularity of lymphatics in the heart, the flow in the capillaries is high, determined by the effects of heart pressure. If lymphatics are obstructed by tumour cells, lymph will stagnate in the myocardial regions upstream of the obstruction, and endocardial to epicardial drainage by the lymphatics will be inhibited. This in turn leads to tissue damage due to lymph stasis, and favours an increased proliferation of neoplastic cells in undrained regions and retrograde lymph flow, which might disseminate malignant cells to the more internal parts. As a result of increased pressure, the lymphatic wall may also break, leading to interstitial tumour spread. The blockade of the common lymphatic node by neoplastic cells coming from metastasised mediastinum lymph nodes is a key event leading to the formation of metastases. The penetration into the myocardium is favoured by the stasis of the lymph flow, and most importantly by the damage to the epicardial plexus caused by the stasis in part, but also by the direct effect of the tumor cells [18,52].

Interestingly, metastatic cell does not disseminate necessarily from the primary tumour. The first step in this process is metastasis to a ‘generalizing site’ such as lungs, liver, and so on (primary level of dissemination). This key disseminating site is the first filter encountered by blood-borne or lymph-borne metastases once they are released from the affected lymph nodes. For a given primary tumour, more than one such key metastatic site may exist. Dissemination of malignant cells then proceeds via secondary metastases from the generalising sites (secondary level of dissemination) [53].

Finally, more recent molecular studies have shown that some forms of malignant tumours, including breast cancer, arise from a small subset of cancer stem cells (CSCs), which are often marked by the cell surface antigens CD133, CD144 and CD24. These cells induce tumour formation and are often resistant to conventional anticancer treatments. The CSCs expresses specific repertoire of surface markers, which allows their differential purification, selectively endowed of tumorigenic capacity as opposed to all other subsets. They sustain the growth of heterogeneous cancer tissues, which recreate the full repertoire of tumour cell populations observed in the primary tumour, and most importantly, they display the main functional hallmarks: self-renewal and differentiation. However, the role of CSCs in multistage cancer progression, particularly with respect to metastasis, has not been well-defined and it has remained partially understood [54-57]. Obviously, better understanding of CSCs as a fundamental component of the metastatic cascade may lead to novel therapeutic strategies [58].

### Clinical manifestations and diagnostic procedures

There is no strong correlation between the extent of cardiac metastases and clinical symptomatology. Some tumours, even with extensive spreading to the heart, produce no symptoms and become evident by incidental findings. Usually, heart involvement is not noticed until after death. In some retrospective studies, only about 10% of patients who died of metastatic disease presented with symptoms or signs related with heart failure [9,59]. The symptomatology and clinical presentation vary, depending more on location than on size and can be classified as extracardiac (constitutional and mechanical), cardiac (pericardial, myocardial and intracavitary) or combined. Constitutional symptoms of pyrexia, arthralgias, rash and so on are mostly caused as a result of cytokine release. Mechanical symptoms (dyspnea, orthopnoea and chest discomfort) result from compression of cardiac chambers or coronary arteries or from pericardial irritation or tamponade caused by growth or effusion/haemorrhage within the pericardium. Myocardial symptoms are caused by arrhythmias due to compression or encroachment on the conduction system. Intracavitary symptoms are caused due to secondary deposits that obstruct valvular function, blood flow or combined, or to tumours that cause tumour fragments to embolize into the systemic circulation or the lungs [18,51]. Intracavitary symptoms may also vary with body position, which can alter haemodynamics. Most patients show symptoms of congestive heart failure, superior vena caval obstruction or embolization [60]. According to the anatomic location of the tumour, some special symptoms and physical findings can be grouped together with regard to pericardial, myocardial and endocardial/heart chamber involvement [61-71].

Pericardial involvement may occur alone or may be associated with metastases elsewhere in the heart. Intrathoracic growths, usually carcinoma of the breast and lungs, may extend directly into the pericardium where the tumour may further invade other parts of the heart. The most feared complications are neoplastic pericardial effusions which can be serous, serosanguineous or bloody, and may lack haemodynamic compromise. They may be mild (and result in no symptoms; in cases of a slow increase in pericardial fluid, the pericardium may stretch and the pericardial effusion can remain asymptomatic for some time) but they are commonly symptomatic and often the first manifestation of a heart metastasis [61]. In all cases of a rapid increase in pericardial fluid, cardiac tamponade can result and necessitate immediate pericardiocentesis [4,8]. Slater *et al*. even reported a case of constrictive pericarditis from metastatic breast cancer to the pericardium [62]. Hypotension, dyspnea, venous congestion, peripheral cyanosis, pulsus paradoxus and fall in QRS voltage are all hallmarks of diagnosis. Chest X-ray may reveal an increase in cardiac silhouette. The electrocardiogram (ECG) may be helpful in demonstrating pericarditis, but as Lamberta *et al*. concluded: ‘The electrocardiographic changes illustrative of pericarditis are minimal in subjects with malignancies of the pericardium unless there is suifficient involvement’ [63]. Tumour-induced pericardial effusion and pericardial masses can be best viewed by means of 2D-TTE and 2D-TEE imaging, CT scan, high resolution CT and MRI. Moreover, the pericardiocentesis specimen can be submitted to cytological analysis to typify the tumour cells and help identify the primary tumour origin.

Myocardial involvement may occur from encroachment on the pericardium, or conduction system by secondary deposits. Tumour tissue in the myocardial mass is usually asymptomatic and does not produce any characteristic ECG signs, but there are many exceptions. Generally, the clinical pattern is proportional to the degree of myocardial infiltration, or related to the wall infiltration site. Whenever the right heart is involved, the interventricular septum is likely to share in the process, and the conduction system may be compromised. Arrhythmias may occur, however, without septal invasion, and they are important for ante mortem diagnosis. Different ECG changes are extremely common in cardiac metastasis and ECG has been of some help [64,65]. Typical presentation includes atrial flutter or fibrillation, premature beats and paroxismal supraventricular and ventricular arrhythmias (commonly tachycardias) conduction disturbances and atrioventricular blocks [64]. Tumour emboli occasionally plug a vessel and produce coronary insufficiency, symptoms of angina pectoris or myocardial infarction [3]. Association between tumour infiltration of the heart and cardiac rupture has been reported only in isolated cases. Metastases to the heart may affect the muscle sufficiently to produce congestive heart failure. Scott and Garvin concluded that the development of congestive failure without apparent cause in patients with malignant disease raises the possibility of cardiac metastases. [8]. Ceardic metastases may also cause T-wave changes, S-T deviations and Q waves [9]. Displacement of the RS-T segment, inverted T waves and isoelectric S-T segments in all leads may also be found [66,67].

Heart chambers involvement is uncommon and the symptoms of venous and intracardiac involvement are often minimal in relation to the extensive involvement seen. Tumour emboli reaching the chambers may implant on the endocardium directly causing valvular stenosis, valvular insufficiency or heart failure. Murmurs typical of stenosis may be caused by metastatic deposits and may change with position. Syncope and sudden death have also been reported [47,68]. Furthermore, intracavitary right-sided heart metastases can lead to pulmonary embolism, and left-sided heart metastases to systemic embolism [50,69,]. Most of the endocardial growths cause little disability and, like most metastatic deposits of the heart, are usually unexpected and incidental necropsy findings. Endocardial metastases may also mimic bacterial endocarditis which is relevant for differential diagnosis. Tumour cells that infiltrate the lumen of a great vein may initiate the deposition of fibrin upon them. The fibrin, in turn, serves as a framework for continued tumour growth. The thrombosis may also extend along the vein to the heart chambers, hindering blood flow and effective heart function. The left atrium may be invaded from the extension of tumour thrombosis of the pulmonary veins. 2D-TTE and 2D-TEE with CT or MRI are valuable in delineating the presence or absence of intracardiac tumour masses [6,50,69]. Nuclear medicine techniques previously employed to detect secondary heart deposits include blood pool imaging and gallium or thallium scintigraphy [70]. Positron-emission tomography computed tomography (^18^F] FDG-PET/CT) has already been proven be more accurate than CT in the evaluation of metastatic heart tumours, especially in the follow-up of patients previously treated with chemotherapy and/or radiotherapy [71]. ^18^F]FDG-PET/CT, positron-emission tomography MRI (PET-MRI) and 3-dimensional echocardiography may have the advantage of detecting the metastases in comparison with other conventional imaging modalities [71-73].

### Treatment

The pathophysiological and clinical aspects of cardiac metastases are intriguing, but, whatever the treatment, their clinical evolution is usually disappointing. In many cases, secondary deposits manifest in patients with advanced cancer disease, with the heart being involved in the generalised cancer spread. Most patients will already have undergone surgical treatment for the primary tumour, or chemotherapy and/or radiation therapy. The treatment of heart metastases depends on tumour origin and patient’s general condition (correlation with Karnofsky and ECOG performance scores), and may include different systemic chemotherapy or palliation [51]. However, the treatment is usually palliative because the prognosis is poor, but radiation therapy can successfully palliate cardiac metastases while preserving quality of life. In the specific cases where secondary cardiac involvement leads to the initial diagnosis of primary tumour, or is the first evidence of metastatic recurrence, aggressive management may result in long-term survival [18,74]. Surgery would be only considered in patients whose primary tumour is under control and who have a significant disease-free interval. Surgery is also indicated in exceptional cases of solitary intracavitary growth, leading to obliteration of heart chambers or valve obstruction, and the patient appears to have a good prognosis [75]. However, in both cases, the postoperative mortality is significant [76,77]. This poor prognosis points to the importance of selecting an appropriate mode of therapy, taking into consideration each patient individually [78].

## Conclusion

Progress in cancer biology has expanded our understanding of the molecular and cellular mechanisms of cancer development, metastatic spreading to the heart and other tissues, as well as resistance of cancer cells to chemotherapy and radiotherapy. This manuscript has reviewed some of the key pathways that have been shown to play an important role in the process of breast cancer spreading to the heart and different diagnostic and treatment modalities. However, breast cancer and other malignant tumours, as a result of their genomic instability, have tremendous redundancy in their ability to maintain growth and to spread into surrounding tissues and distant organs. As such, this remarkable level of complexity makes successful treatment of breast cancer metastases all the more challenging. According to some reports, an average survival period after the diagnosis of cardiac metastases has been approximately 5.5 months [77]. Obviously, it is very important to know that in cancer patients with symptoms of heart failure, metastatic cardiac deposits must always be considered in the differential diagnosis, even in the subjects with a distant positive medical history. Clinicians should be alert to the possibility of cardiac metastasis in a patient with and even without clinical symptoms. 2D-TTE and 2D-TEE, which are easy, fast and sensitive techniques for detection of cardiac metastasis, and CT, MRI, ^18^F]FDG-PET/CT and PET-MRI as confirming methods should always be performed when any suspicion exists in order to exclude cardiac involvement during clinical follow-up [65,67,71-73,77]. This case also demonstrates that complete mastectomy and other oncological and surgical procedures may result in a favourable outcome for many years, but regular medical lifelong follow-up is mandatory.

## Consent

Written informed consent was obtained from the patient for publication of this case report and any accompanying images. A copy of the written consent is available for review by the Editor-in-Chief of this journal.

## Abbreviations

[^18^F]FDG-PET/CT: 18F-fluorodeoxyglucose-positron-emission-tomography-computed tomography; 2D-TEE: Two-dimensional transesophageal echocardiography; 2D-TTE: Two-dimensional transthoracic echocardiography; BM: Basement membrane; Ca 15–3: Carbohydrate antigen 15–3; CEA: Carcinoembryonic antigen; CD133: Cluster of differentiation 133; CD144: Cluster of differentiation 144; CD24: Cluster of differentiation 24; CMF: Cyclophosphamide, methotrexate and fluorouracil; CSCs: Cancer steam cells; CT: Computed tomography; ECG: Electrocardiogram/electrocardiography; ECM: Extracellular matrices; ECOG PS: Eastern cooperative cncology group performance status scale; EMT: Epithelial to mesenchymal transition; ER: Estrogen receptor; Gy: Gray; HER-2/neu: Human epidermal growth factor receptor 2; KPS: Karnofsky’s index of performance status; MSCT: Multislice computed tomography; MMP: Matrix metalloproteases; MRI: Magnetic resonance imaging; NF-κB: Nuclear factor kappa-light-chain-enhancer of activated B cells; NPC: Nuclear pore complex; Nup88: Nucleoporin 88; Nups: Nucleoporins; PET-MRI: Positron-emission tomography-magnetic resonance imaging; PHD: Pathohistological diagnosis; Rae1: Ribonucleic acid export 1; TGF-β: Transforming growth factor-β; TNC: Tenascin C.

## Competing interests

Each co-author certifies that s/he has no commercial association that might pose a conflict of interest in connection with the submitted article.

## Authors’ contributions

DK, RSP and SP carried out the study design and writing. II, IA and NN participated in the data collection and interpretation and drafted the manuscript. DK, RSP, FS and II participated in the design of the study and the figure design. SP, DT, II, AJ and NN participated in the literature search and helped to draft the manuscript. All authors have read and approved the final manuscript.
